# Regulation of TGF-β and BMP Signaling by Natural Triterpene Compounds in Pulmonary Arterial Hypertension (PAH)

**DOI:** 10.3390/cimb47110939

**Published:** 2025-11-12

**Authors:** Sila Ozlem Sener, Sabita Shaha, Saltan Gülçin İşcan, Ufuk Ozgen, Merve Yuzbasioglu Baran, Aleyna Nalcaoğlu, Md Talat Nasim

**Affiliations:** 1 Department of Pharmacognosy, Gulhane Faculty of Pharmacy, University of Health Sciences, Ankara 06018, Türkiye; silaozlem.sener@sbu.edu.tr (S.O.S.); merve.yuzbasioglu@sbu.edu.tr (M.Y.B.); 2 Translational Medicine Laboratory, School of Pharmacy, Optometry and Medical Sciences, University of Bradford, Bradford BD7 1DP, UK; s.shaha2@bradford.ac.uk; 3 Department of Pharmacognosy, Faculty of Pharmacy, Ankara University, Ankara 06100, Türkiye; gulcin.saltan@pharmacy.ankara.edu.tr; 4 Department of Pharmacognosy, Faculty of Pharmacy, Karadeniz Technical University, Trabzon 61080, Türkiye; uozgen@ktu.edu.tr; 5 Department of Molecular Biology and Genetics, Faculty of Science, Karadeniz Technical University, Trabzon 61080, Türkiye; aleynanalcaoglu@ktu.edu.tr

**Keywords:** BMPR-II, lupeol, ψ-taraxasterol, PAH, TGFBR2

## Abstract

Pulmonary arterial hypertension (PAH) is a devastating cardiovascular disorder caused by right heart failure leading to premature death. The TGFBR2 and BMPR-II receptors, which are members of the TGF-β receptor family, are considered promising targets for developing novel drugs in PAH. Lupeol and ψ-taraxasterol, naturally occurring triterpene molecules with proven anti-inflammatory, anti-cancer, and cardioprotective activities, hold considerable potential in the treatment of PAH. Hence, the present study aimed to evaluate the impacts of lupeol and ψ-taraxasterol isolated from *Cirsium sintenisii* Freyn on the TGF-β and BMP pathways, aiming to determine their therapeutic values in PAH. The effects of the compounds were extensively investigated using both in silico and wet lab experiments, including reporter assays, RT-PCR/QPCR, Western blots, and cell proliferations assays. Both lupeol and ψ-taraxasterol demonstrated interactions with the majority of components of these signaling pathways, including the TGFBR2 and BMPR-II receptors, suggesting that both compounds were capable of modulating the BMP and TGF-β pathways. Data derived from reporter assays, RT-PCR/QPCR, and Western blots demonstrated that lupeol and ψ-taraxasterol inhibited the TGF-β signaling pathway by reducing the phosphorylation of the SMAD3 protein and the expression of *pai*-1 transcripts. Additionally, ψ-taraxasterol enhanced BMP signaling via regulating the phosphorylation of SMAD1/5 proteins and upregulated the expression of *id*-1 transcripts. Finally, lupeol and ψ-taraxasterol inhibited abnormal proliferation of mutant-type (*bmpr2*^R899X+/-^) PAMSCs stimulated with the TGF-β1 ligand with no discernible effects on wild-type cells. This is the first comprehensive report outlining the potential therapeutic effects of lupeol and ψ-taraxasterol in PAH, which may have immediate experimental and clinical applications not only in PAH but also other BMP- and TGF-β-associated disorders.

## 1. Introduction

Pulmonary hypertension (PH) is a pathophysiological condition characterized by a variety of clinical manifestations and is defined by a mean resting pulmonary artery pressure of ≥25 mmHg. Pulmonary arterial hypertension (PAH), one of the forms of PH, is described by a resting mean pulmonary artery pressure ≥ 25 mmHg, a normal pulmonary artery wedge pressure ≤ 15 mmHg, and a pulmonary vascular resistance > 3 Wood’s units [1]. PAH is a serious vascular disease characterized by the constriction and blockage of small pulmonary arteries due to the abnormal proliferation of pulmonary smooth muscle cells (PAMSCs). In the absence of adequate treatment, pulmonary vasoconstriction and vascular remodeling associated with pulmonary arterial hypertension (PAH) lead to elevated pulmonary artery pressure, right ventricular failure, and potentially fatal outcomes [2]. PAH currently lacks a definitive curative treatment. The survival rate of PAH patients over a 5-year period ranges from 49% to 67% with the utilization of existing therapeutic interventions. Currently, drugs used in the treatment of PAH are insufficient as they are unable to reduce the progression of vascular and cardiac remodeling. Hence, innovative and efficient treatments focusing on the fundamental molecular pathways involved in pulmonary vascular remodeling are necessary for the management of PAH [2,3].

Familial PAH is primarily caused by mutations in the *BMPR2* gene [4]. The bone morphogenic genetic protein receptor II (BMPR-II) is a member of the transforming growth factor-β (TGF-β) superfamily. Mutations have also been found in SMADs namely SMAD1, 4, and 9 [5]. Heterozygous mutations have been identified in 70% of individuals with hereditary PAH and 25% of individuals with idiopathic PAH. Nonsense mutations in the *BMPR2* gene cause a significant reduction in BMPR-II protein expression in idiopathic and hereditary PAH and PASMCs isolated from PAH patients [6,7,8]. PAH causes remodeling of pulmonary vessels due to a lack of BMPR-II expression in PASMCs. Hence, the migration and excessive proliferation of PASMCs in PAH are directly correlated with deficiencies in the BMP signaling pathway [6,9].

Earlier on, we demonstrated that the insufficiency of the BMP signaling pathway results in heightened and atypical stimulation of the TGF-β signaling pathway [10]. It was also demonstrated that the TGF-β pathway is activated in PAH patients and *in vivo* PAH rat lungs [6,11]. Preclinical investigations demonstrated that in PAH, elevated TGF-β signaling causes smooth muscle hypertrophy, perivascular fibrosis, and remodeling of the extracellular matrix. The results of these studies indicate that an increase in TGF-β signaling results in a pro-proliferative response in PAMSCs. In addition, the mutant *BMPR2* promotes excessive proliferation of PAMSC’s harboring a pathogenic *BMPR*2 mutation via a high level of TGF-β ligand secretion. The data unequivocally indicates that dysregulated TGF-β signaling is responsible for vascular remodeling and contributes to the pathogenesis of both clinical and preclinical cases of PAH [12,13].

TGF-β ligand binding to the TGFβR2 causes the activation of the TGFβR2 kinase of the receptor, which directly phosphorylates the TGFβR1 receptor, leading to the phosphorylation relay of R-SMADs (SMAD2/3). Phosphorylated R-SMADs and SMAD4 form a complex after dissociating from type I receptors. Similarly for the BMP pathway, the BMP ligands form a complex that first phosphorylates BMPR-II receptor kinase and then activates BMPR-I. The activated receptor complex activates R-SMADs (SMAD 1/5/8) [13,14,15].

We and others reported that the expression of TGF-β superfamily target genes, namely plasminogen activator inhibitor-1 gene (*pai*-1) and inhibitor of the protein DNA binding-1 gene (*id*-1), are altered in PAH [6]. The plasminogen activator inhibitor-1 gene (*pai*-1) is one of the major transcriptional targets of the TGF-β signaling pathway. *pai*-1 is stimulated by the complex of SMAD2/3 and SMAD4 [16,17]. The inhibitor of the protein DNA binding-1 gene (*id*-1) is the major transcriptional target of the BMP signaling pathway. The regulation of BMP-dependent gene transcription in PAMSC is contingent upon the coordination of SMAD activity. Mutant *bmpr2* reduces the activation of SMAD1/5 in PAMSCs, leading to a reduction in the *id*-1 transcription level. The disruptions in *pai*-1 and *id*-1 expression levels in PAMSCs contribute to abnormal proliferation and differentiation, leading to the progression of PAH [18,19].

Natural products are important sources for the discovery of new lead compounds in drug development research. Natural compounds have been utilized as active ingredients in the treatment of various diseases like cancer, diabetes, cardiovascular diseases, and metabolic syndrome, and they also serve as the first molecules used to develop synthetic and semi-synthetic drug molecules [20]. Despite the limited research on natural compounds in PAH treatment, they have become increasingly popular in recent years [4,21,22].

*Cirsium* species, derived from the Greek name “khirsos” meaning “swollen veins”, have been used in circulatory system disorders traditionally. The hypertension-related effects of *Cirsium* species, such as anti-inflammatory, venoactive, diuretic, antihypertensive, and antiobesity, have been proven [23]. Several studies have investigated the potential of *Cirsium* species in the management of hypertension. Among them, the antihypertensive activity of *Cirsium japonicum* has been particularly well documented. The aqueous extract of this species has been reported to exert significant antihypertensive effects by inducing vasorelaxation in isolated rat thoracic aorta pre-contracted with norepinephrine, a response mediated through H_1_ histamine receptor activation. This effect has been associated with elevated levels of nitric oxide (NO) and cyclic guanosine monophosphate [24]. Furthermore, in a two-kidney, one-clip renal hypertension model, *Cirsium japonicum* demonstrated cardioprotective properties by increasing serum NO and nitric oxide synthase (NOS) levels while simultaneously reducing plasma angiotensin II concentrations [25]. Therefore, *Cirsium* species have the potential to be used for hypertension.

Triterpenes constitute a large group of compounds of natural origin, which are formed by the arrangement of squalene epoxide in the form of chair–chair–chair–boat and subsequent condensation. There is increasing interest in triterpenes due to their diverse biological activities [26]. Lupeol is a naturally derived compound with a pentacyclic lupane-type triterpene structure. Many studies have revealed that lupeol has many pharmacological activities, such as antioxidant, anti-inflammatory, antihyperglycemic, antihyperlipidemic, and antimutagenic effects. Various administration routes, such as topical, oral, subcutaneous, intraperitoneal, and intravenous, have been utilized in *in vivo* studies to investigate the effects of lupeol. These studies have demonstrated the compound’s efficacy in treating conditions such as diabetes, asthma, and arthritis, as well as its protective effects on the heart, liver, kidneys, and nervous system. Additionally, lupeol has shown promise regarding its anti-cancer properties [27]. ψ-taraxasterol contains a pentacyclic triterpene structure like lupeol and has the same molecular formula. Unlike lupeol, ψ-taraxasterol, which is an isomer of lupeol, contains a taraxastrene-type ring system instead of lupen [28,29,30]. Oral administration of 40 mg/kg of a triterpene mixture containing ψ-taraxasterol to rats reduced acetic acid-induced pain. The same mixture also inhibited peritoneal leukocyte infiltration in a carrageenan-induced inflammation model in mice and produced an anti-inflammatory effect [31].

In this study, lupeol and ψ-taraxasterol were isolated from *Cirsium sintenisii Freyn.* Next, we explored the effect of lupeol and ψ-taraxasterol on PAH by identifying the underlying molecular mechanism of TGF-β and BMP pathway regulation. We found that both compounds modulate both TGF-β and BMP pathways and may have experimental and clinical applications for the resolution of PAH and other TGF-β- and BMP-associated disorders.

## 2. Materials and Methods

### 2.1. Isolation Studies

***Plant material***

The aerial parts *Cirsium sintenisii* Freyn were collected from Antalya, Türkiye (1766 m, 17 August 2017). The voucher specimens were deposited at the Herbarium of Faculty of Education, Balikesir University (Balıkesir, Turkey), with voucher number “Dirmenci 4917”.

***Isolation of compounds***

The scheme of the isolation procedure is stated below (Figure S1).

***Identification of compounds***

The molecular structures of the active compounds were elucidated through the application of Nuclear Magnetic Resonance (NMR) spectroscopy and Mass Spectrometry (MS). The compounds were solubilized in deuterated methanol (CD_3_OH) or deuterated chloroform (CDCl_3_), and NMR spectra were recorded utilizing a Bruker Ascend 400 MHz NMR spectrometer (Bruker Corporation, Billerica, MA, USA). Mass spectrometric analyses were performed with a Waters Micromass^®^ ZQ mass spectrometer (Waters Corporation, Milford, CT, USA).

### 2.2. Cell Culture, Plasmid DNA Isolation, and Transfection Procedure

The cell culture and transfection procedure were carried out as previously described [32,33]. Mutant-type PASMCs obtained from knock-in mice (*bmpr2*^R899X+/-^) harboring a mutation in the *bmpr2* gene, wild-type PAMSCs (*bmpr2*^+/+^) derived from wild-type mice, and HEK 293T cells (ATCC, Manassas, VA, USA; (database: Cellosaurus; accession number: CVCL_0063)) were used for cell culture studies. The cells were grown in Dulbecco’s Modified Eagle’s Medium (DMEM) (ThermoFisher Scientific^®^, Waltham, MA, USA) supplemented with 10% Fetal Bovine Serum (FBS) (Merck^®^, Darmstadt, Germany), 1% Penicillin/Streptomycin (Pen/Strep) (Merck, Rahway, NJ, USA), and 1% L-glutamine (Merck, USA). Plasmids that were used for the transfection process were isolated using the QIAprep Spin Miniprep kit (QIAGEN^®^, Hilden, Germany). The transfection procedure was conducted using Gene Jammer Transfection reagents employing the plasmids encoding TGFβR2 (100 ng/mL), SBE-LUC (100 ng/mL), and ΒGAL (50 ng/mL) for the TGF-β pathway following the manufacturer’s protocols. To investigate the BMP pathway, a similar transfection procedure was carried out with the exception of plasmids of BMPR-II (100 ng/mL), 3-GC (100 ng/mL), and BGAL (50 ng/mL). Following the transfection process, 0.1–20 µM concentrations of the natural compounds diluted with 0.1% serum-free Dulbecco’s Modified Eagle’s Medium (DMEM) (ThermoFisher Scientific^®^, Waltham, MA, USA) were used for the drug treatment procedure.

### 2.3. Western Blotting

The Western blot analysis was carried out following the methodology established by Nasim and colleagues [32]. The antibodies of phosphor SMAD1/5 ((Cell Signaling Technology^®^, Danvers, MA, USA)) for the BMP pathway and phosphor SMAD3 (Cell Signaling, MA) for the TGF-β pathway were used. The membranes were reprobed using a β-actin antibody (Cell Signaling, MA) to ensure equivalent loading. The membrane was positioned on a level surface, and the ECL prime Western blot detection reagent (GERPN2236) was applied. Images of the membranes were acquired using the Chemidoc Imaging System (Bio-Rad^®^, Hercules, CA, USA).

### 2.4. SMAD-Responsive Reporter Assay

The SMAD-responsive reporter assay was conducted in transfected HEK 293T cells following established protocols [32]. The Dual-Light Reporter Assay systems from Applied Biosystems^®^, Foster City, CA, USA, were employed with an ORION-II Plate Luminometer from Titertek, Berthold Technologies^®^, Bad Wildbad, Germany, as per the manufacturer’s guidelines for quantifying luciferase and β-galactosidase activities.

### 2.5. RT-PCR Analysis

The protocol for isolating RNA from transfected HEK293T cells involved following the Ambion-PureLink RNA mini kit procedure [10]. In order to obtain cDNA from RNA, a reaction mixture was prepared by combining 14 μL of diluted RNA and 1 μL of random primer (Agilent Technologies^®^, Santa Clara, CA, USA) in reaction mixture 1 and 5× M-MLV Reverse Transcriptase Buffer (Thermofisher Scientific, USA), 25 mM dNTP (Thermofisher Scientific, USA), 20 U RNasin (Thermofisher Scientific, USA), 100U M-MLV Reverse Transcriptase (Thermofisher Scientific^®^, Waltham, MA, USA), and RNase free water (Thermofisher Scientific, USA) in reaction mixture 2. The negative control tube was assembled using identical components, with the exception of M-MLV Reverse Transcriptase. All PCR tubes were incubated at 37 °C for 1 h in a thermocycler machine to synthesize cDNA. PCR master mix (12.5 µL Dream TaqMan mix (Thermofisher Scientific, USA), with 2 µL of the Forward Primer of pai-1 (Thermofisher Scientific, USA), 2 µL of the Reverse Primer of pai-1 (Thermofisher Scientific, USA), and 0.5 µL of nuclease-free water (Thermofisher Scientific, USA), 8 µL cDNA sample), was prepared. β-actin was used as a housekeeping gene for an internal standard. After adding the mixture and loading the dye (SYBR-Safe), the tubes were transferred into a thermocycler and run for an hour and a half. The agarose gel in the RT-PCR equipment was loaded with related samples. After loading samples, the agarose system was run for 1 h at 120 V and 400 mA. The gel was imaged using the Chemidoc Imaging System (Bio-Rad, Watford, UK).

### 2.6. Q-PCR Analysis

The method described in a previous study was employed for conducting Q-PCR Analysis [10]. A PCR master mix was prepared, consisting of 250 µL of Taq master mix (Applied Biosystems, Carlsbad, CA, USA), 25 µL of the probe of id-1 (Applied Biosystems, USA) or pai-1 (Applied Biosystems, USA), and 175 µL of RNase-free water (Applied Biosystems, USA). PCR master mix and diluted cDNA samples were added to the relevant well of the 96-well clear PCR plate. The plate was run with the ™StepOne™ Real-Time PCR System Software program for 78 min by a PCR machine (Applied Biosystems, USA). The samples were incubated at 95 °C for 15 min during the analysis, followed by 40 cycles of denaturation at 95 °C for 15 s and a final extension and annealing step at 60 °C for 60 s.

### 2.7. Proliferation Assay

The MTT assay utilized a protocol that closely resembled the one outlined by Ogo et al. in 2013 and Lento et al. in 2015 [10,34]. Mutant-type (*bmpr2*^R899X+/-^) and wild-type (*bmpr2*^+/+^) PAMSCs were plated at a concentration of 0.1 × 10^5^ cells per well in 96-well microplates with Dulbecco’s Modified Eagle’s Medium (DMEM) supplemented with 10% FBS, 1% Pen/Strep, and 1% L-glutamine. The natural compounds were diluted with 10ng/mL of TGF-β1 ligand solution of colorless 0.1% DMEM media for drug treatment. Cell proliferation was evaluated according to the manufacturer’s instructions utilizing the Cell-Titer 96 Aqueous One Solution Cell Proliferation Assay from Promega in Madison, WI. The wells’ absorbance was quantified at a wavelength of 510 nm utilizing the iMark™ Microplate Absorbance Reader (Bio-Rad Laboratories^®^, Hercules, CA, USA) through spectroscopic analysis.

### 2.8. Calculations and Statistical Analysis

The experiments were repeated a minimum of three times. Statistical analysis was performed with GraphPad Prism 9.4.0 [35]. The statistical significance of the differences between the data was presented as means ± standard deviations (SDs). The data were analyzed using one-way ANOVA, followed by the Tukey post hoc test (APA-(ns-0.12) (* 0.033) (** 0.002) (*** < 0.001) for comparing multiple means.

### 2.9. In Silico Analysis

AutoDock 1.5.7 software was utilized to conduct molecular docking calculations in order to evaluate the interaction between the synthesized compound and target proteins. The structures of the target proteins were obtained from the Protein Data Bank (www.rcsb.org, accessed on 29 September 2025). Specifically, the C domain of the 7PPA structure from homo sapiens with a resolution of 1.48 Å was used for the extracellular domain of BMPR-II. On the other hand, the extracellular domain of the TGFBR2 receptor utilized the receptor 4XJJ (Resolution: 1.40 Å, Chain: A) from the Protein Data Bank as a reference. The compounds lupeol and ψ-taraxasterol were sourced from the PubChem database. The UFF force field was used with Avogadro 2.0 software to optimize the ligands [36,37]. To save the receptors in the PDBQT format for docking, water molecules, ions, and other ligands were eliminated, and polar hydrogens and the Kollman charge were added. During the docking, the ligands were allowed to be flexible. The receptor molecules were encompassed by a grid, which was measured in the x, y, and z directions, with a spacing of 0.420 Å. To perform the docking, the Lamarckian genetic algorithm method was employed, with a population size of 150 individuals and a maximum of 2,500,000 energy evaluations. Result files were created in AutoDock 1.5.7. Software and the binding poses and energy calculations of the docked structures were visualized and analyzed using BIOVIA Discovery Studio Visualizer [38].

## 3. Results

### 3.1. Isolation of Triterpene Compounds

**Figure 1:**
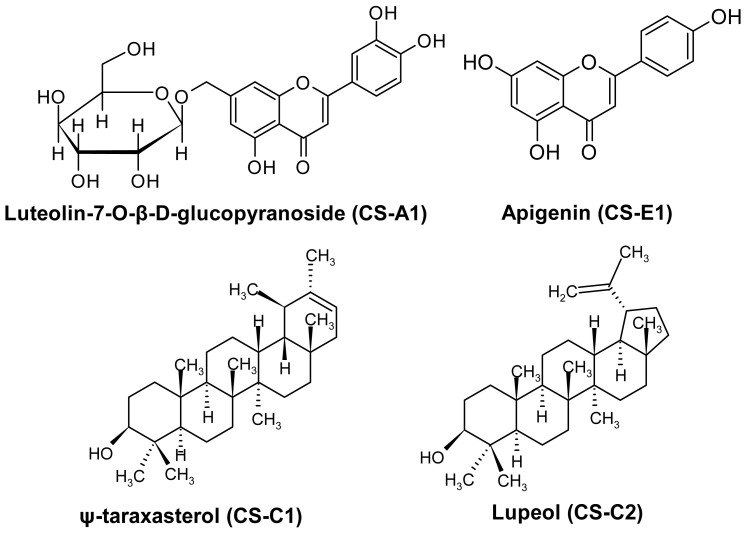
Molecular structure of isolated compounds.

Isolation studies led to the purification of compounds as two flavonoids, luteolin-7-O-β-D-glucopyranoside (CS-A1) and apigenin (CS-E1), and two triterpenoids, ψ-taraxasterol (CS-C1) and lupeol (CS-C2) (Figure 1).

CS-A1 was displayed as an *m*/*z*: 609.12 [M − H]^+^ (C_27_H_30_O_16_) ion according to MS analysis. ^1^H-NMR (CD_3_OD, 400 MHz): δ 7.45 (*d*, *J* = 2.2 Hz, H-6′), 7.43 (bs, H-2′), 6.92 (*d*, *J* = 8.28 Hz, H-5′), 6.62 (*s*, H-3), 6.82 (1H, *d*, *J* = 2.1, H-8), 6.51 (1H, *d*, *J* = 2.0, H-6), 5.15 (1H, *d*, *J* = 7.2, H-1″), 4.02 (1H, *d*, *J* = 9.3, H-2″), 3.32–3.61 (m, H-3″, H-4″, H-5″, H-6″). CS-A1 was identified as the flavonol glycoside luteolin-7-O-β-D-glucopyranoside. ^1^H-NMR data of the compounds were compatible with the published literature (Figure S2) [39].

CS-E1 was displayed as an *m*/*z*: 274.27 [M − H]^+^ (C_15_H_5_O_5_) ion according to MS analysis. ^1^H-NMR (CD_3_OD, 400 MHz): δ 7.86 (*d*, *J* = 7.9 Hz, H-2′, H-6′), 6.94 (*d*, *J* = 8.1 Hz, H-3′, H-5′), 6.60 (*s*, H-3), 6.45 (*s*, H-8), 6.22 (*s*, H-6). ^13^C-NMR (CD_3_OD, 100 MHz): δ 182.5 (C-4), 164.9 (C-7), 164.7 (C-2), 161.8 (C-4′), 161.4 (C-5), 158.0 (C-9), 128.1 (C-2′, C-6′), 121.8 (C-1′), 115.6 (C-3′, C-5′), 103.7 (C-3), 102.4 (C-10), 98.7 (C-6), 93.6 (C-8). CS-E1 was identified as the flavonol apigenin. ^1^H-NMR and ^13^C-NMR data of the compounds were compatible with the published literature (Figure S3) [40,41].

CS-C1 gave an *m*/*z*: 427.32 [M + H]^+^ (C_30_H_50_O) ion. ^1^H-NMR (CDCl_3_, 400 MHz): δ 5.18 (*d*, *J* = 6.7 Hz, H-21), 3.13 (*dd*, *J*_1_ = 11.3 Hz, *J*_2_ = 5.0 Hz, H-3), 0.86–1.66 (m, H-1, H-2, H-5, H-6, H-7, H-9, H-11, H-12, H-13, H-15, H-16, H-18, H-19, H-22), 1.55 (s, H-30), 0.96 (s, H-26), 0.91 (*bs*, H-29), 0.90 (*s*, H-23), 0.87 (*s*, H-27), 0.77 (*s*, H-25), 0.69 (*s*, H-24), 0.66 (*s*, H-28); ^13^C-NMR (CDCl3, 100 MHz): δ 139.9 (C-20), 118.9 (C-21), 79.0 (C-3), 55.3 (C-5), 50.4 (C-9), 48.7 (C-18), 42.3 (C-22), 42.2 (C-14), 41.1 (C-8), 39.2 (C-13), 38.9 (C-4), 38.8 (C-1), 37.1 (C-10), 36.7 (C-16), 36.3 (C-19), 34.4 (C-17), 34.2 (C-7), 28.0 (C-23), 27.6 (C-12), 27.4 (C-2), 27.0 (C-15), 22.6 (C-29), 21.7 (C-30), 21.6 (C-11), 18.3 (C-6), 17.7 (C-28), 16.3 (C-25), 16.0 (C-26), 15.4 (C-24), 14.7 (C-27). CS-C1 was detected as ψ-taraxasterol based on the ^1^H-NMR and ^13^C-NMR data of the compound (Figure S4) [42].

CS-C2 was displayed as an *m*/*z*: 425.51 [M − H]+ (C_30_H_50_O) ion according to MS analysis. ^1^H-NMR (CDCl_3_, 400 MHz): δ 4.70 (*bs*, H-29b), 4.58 (*bs*, H-29a), 3.21 (*dd*, *J*_1_ = 11.2 Hz, *J*_2_ = 4.9 Hz, H-3), 2.40 (sextet, *J*_1_ = 11.0 Hz, *J*_2_ = 5.8 Hz, H-19), 2.20 (*m*, H-21a), 0.69–1.70 (*m*, H-1, H-2, H-5, H-6, H-7, H-9, H-11, H-12, H-13, H-15, H-16, H-18, H-22), 1.70 (*s*, H-30), 1.40 (*m*, H-21b), 1.05 (*s*, H-26), 0.98 (*s*, H-23), 0.96 (*s*, H-27), 0.85 (*s*, H-25), 0.80 (*s*, H-28), 0.78 (*s*, H-24); ^13^C-NMR (CDCl_3_, 100 MHz): δ 151.0 (C-20), 109.4 (C-29), 79.0 (C-3), 55.3 (C-5), 50.4 (C-9), 48.3 (C-18), 48.0 (C-19), 43.0 (C-17), 42.8 (C-14), 40.8 (C-8), 40.0 (C-22), 38.9 (C-4), 38.7 (C-1), 38.0 (C-13), 37.2 (C-10), 35.6 (C-16), 34.3 (C-7), 29.8 (C-21), 28.0 (C-23), 27.4 (C-2), 27.4 (C-15), 25.1 (C-12), 20.9 (C-11), 19.3 (C-30), 18.3 (C-6), 18.0 (C-28), 16.1 (C-25), 16.0 (C-26), 15.4 (C-24), 14.6 (C-27). The compound was confirmed as lupeol in comparison to previous articles (Figure S5) [43].

### 3.2. Determination of Lupeol and ψ-Taraxasterol Interactions with the Components of BMP and TGF-β Pathways Using In Silico Analysis

**Figure 2:**
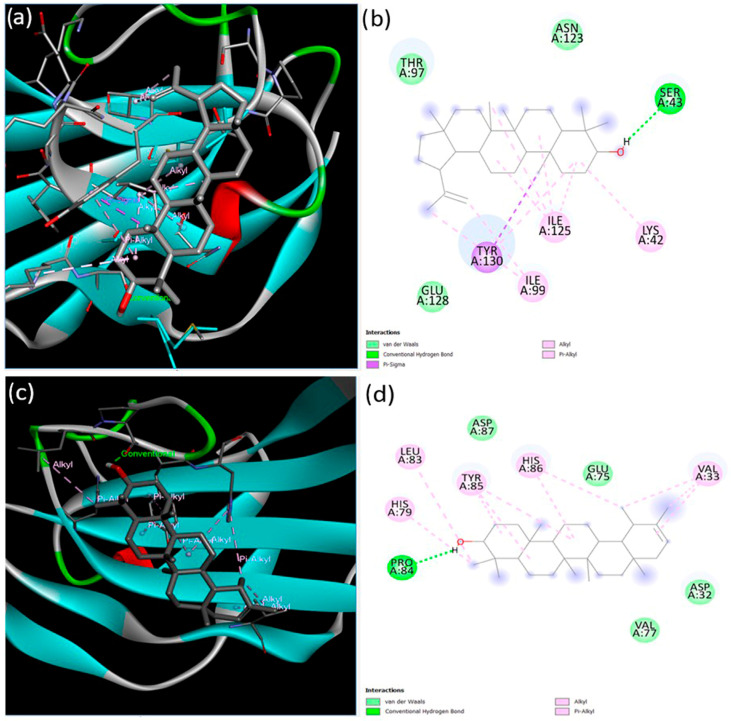
Binding configurations predicted by the BIOVIA Discovery Studio Visualizer with the target TGFBRII extracellular domain receptor. (**a**) The 3D view and (**b**) 2D ligand interactions of lupeol; (**c**) the 3D view and (**d**) 2D ligand interactions of ψ-taraxasterol.

The interactions of lupeol and ψ-taraxasterol with the extracellular domains of TGFBR2 and BMPR-II were investigated. The interaction between TGFBR2 and lupeol was characterized by a minimum binding energy of −7.87 kcal/mol and an estimated inhibition constant of 1.71 microMolar Ki (Figure 2). Detailed examination of the interactions revealed the presence of a single conventional hydrogen bond, seven alkyl bonds, one pi–alkyl bond, one pi-sigma bond, and three van der Waals forces. The strongest interaction was a conventional hydrogen bond at the Ser43 residue, measuring 2.23 Å in length. In contrast, ψ-taraxasterol interacts with the extracellular domain of TGFBRII; a minimum binding energy of −5.17 kcal/mol and an inhibition constant (Ki) of 162.60 µM (micromolar) were observed. In this case, the strongest interaction occurred again through a hydrogen bond at residue Pro84 with a bond length of 1.94 Å. Additionally, six pi–alkyl, four alkyl bonds, and four van der Waals interactions were identified (Figure 2).

**Figure 3:**
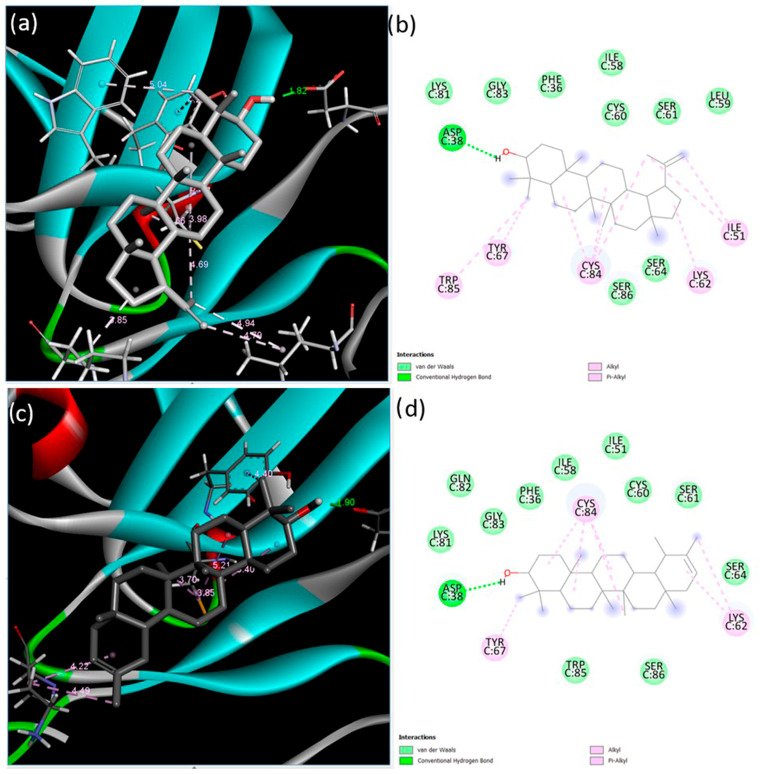
Binding poses predicted by the BIOVIA Discovery Studio Visualizer with the target BMPR-II extracellular domain receptor. (**a**) The 3D view and (**b**) 2D ligand interactions of lupeol; (**c**) the 3D view and (**d**) 2D ligand interactions of ψ-taraxasterol.

Next, the interactions of the extracellular domain of BMPR-II with lupeol and ψ-taraxasterol were examined. The lowest binding energy and estimated inhibition constant value were determined as −9.03 kcal/mol and 238.94 nM (nanomolar) and −8.83 kcal/mol and 334.09 nM (nanomolar) for lupeol and ψ-taraxasterol, respectively. For lupeol-BMPR-II interactions, one conventional hydrogen bond, seven alkyl bonds, two pi–alkyl bonds, and nine van der Waals interactions were identified. The most significant interaction occurred at the Asp38 residue with a length of 1.82 Å. For BMPR-II and ψ-taraxasterol interactions, five alkyl, one pi–alkyl, eleven van der Waals, and one conventional hydrogen bond were observed. The strongest interaction among these bonds occurred at the Asp38 residue with a length of 1.90 Å in the conventional hydrogen bond (Figure 3).

**Figure 4:**
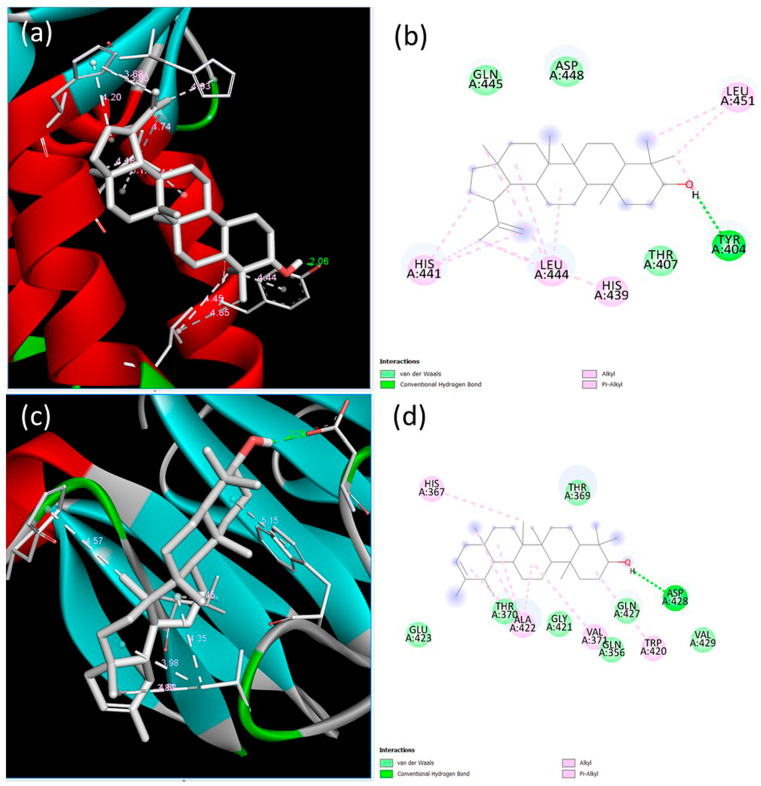
Binding poses predicted by the BIOVIA Discovery Studio Visualizer with the target SMAD1 extracellular domain receptor. (**a**) The 3D view and (**b**) 2D ligand interactions of lupeol; (**c**) the 3D view and (**d**) 2D ligand interactions of ψ-taraxasterol.

BMPR-II conveys signaling via the SMAD1/5/8-mediated pathway, and hence the interactions of the compounds with SMAD1 were investigated. The lowest binding energy and estimated inhibition constant values were determined to be −7.43 kcal/mol and 3.57 µM (micromolar) for lupeol and −8.39 kcal/mol and 705.41 nM (nanomolar) for ψ-taraxasterol, respectively. The lupeol–SMAD1 interactions indicated the presence of one conventional hydrogen bond, two alkyl bonds, two pi–alkyl bonds, and three van der Waals forces. A key interaction was observed at the Tyr404 residue, involving a conventional hydrogen bond with a length of 2.06 Å. Conversely, the interaction between SMAD1 and ψ-taraxasterol displayed two alkyl bonds, two pi–alkyl bonds, seven van der Waals interactions, and one conventional hydrogen bond. The most prominent interaction among these was recorded at the Asp428 residue, with a hydrogen bond length of 2.25 Å (Figure 4).

**Figure 5:**
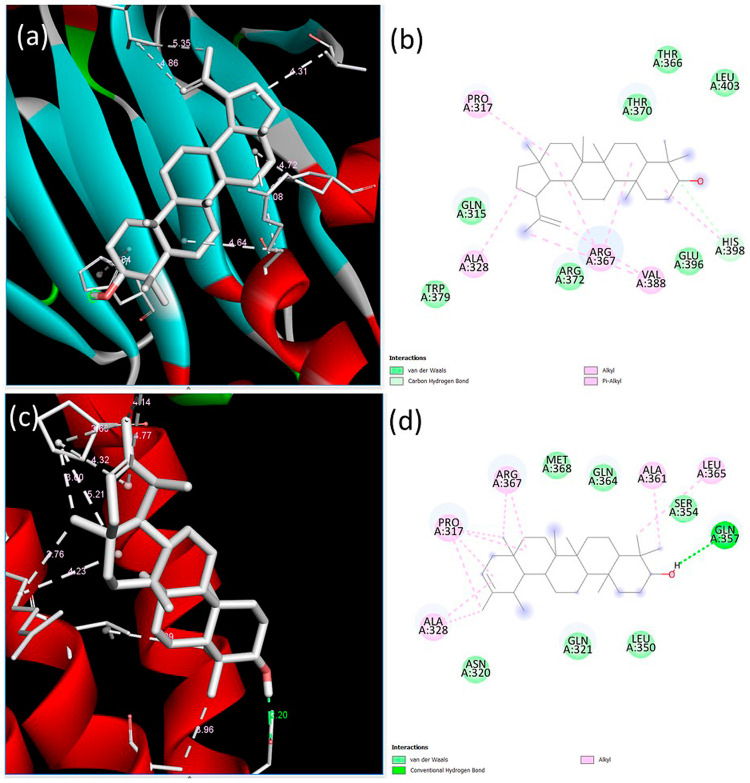
Binding poses predicted by the BIOVIA Discovery Studio Visualizer with the target SMAD3 extracellular domain receptor. (**a**) The 3D view and (**b**) 2D ligand interactions of lupeol; (**c**) the 3D view and (**d**) 2D ligand interactions of ψ-taraxasterol.

Upon examining the interactions between the compounds and SMAD3 protein, the lowest binding energy and estimated inhibition constant values were found to be −6.59 kcal/mol and 14.78 µM (micromolar) for lupeol and −7.80 kcal/mol and 1.93 µM (micromolar) for ψ-taraxasterol, respectively. The interactions between SMAD3 and lupeol revealed one carbon–hydrogen bond, four alkyl bonds, one pi–alkyl bond, and seven van der Waals interactions, with the most prominent interaction occurring at the His398 residue via a carbon–hydrogen bond measuring 3.47 Å. In contrast, the interaction between SMAD3 and ψ-taraxasterol exhibited five alkyl bonds, six van der Waals interactions, and one conventional hydrogen bond, with the most significant interaction occurring at the Gln357 residue, which measured 2.20 Å in the conventional hydrogen bond (Figure 5).

Next, the interactions between the compounds and SMAD4 were investigated. The lowest binding energy and the calculated inhibition constant were determined to be −7.24 kcal/mol and 4.95 µM (micromolar) for lupeol and −8.31 kcal/mol and 811.02 nM (nanomolar) for ψ-taraxasterol, respectively. The SMAD3 and lupeol interactions identified one Pi–sigma bond, one conventional hydrogen bond, three alkyl bonds, two pi–alkyl bonds, and two van der Waals interactions. A notable interaction was observed at the Glu321 residue, characterized by a bond length of 3.00 Å. Conversely, the interaction between SMAD4 and ψ-taraxasterol displayed three alkyl bonds, two pi–alkyl bonds, one pi–sigma bond, five van der Waals interactions, and one conventional hydrogen bond. The strongest interaction among these was observed at the Glu321 residue, with hydrogen bond lengths of 2.68 Å and 2.98 Å (Figure S6).

Subsequently, the interactions between SMAD5 and the compounds lupeol and ψ-taraxasterol were determined. The lowest binding energy and estimated inhibition constant values were found to be −6.91 kcal/mol and 8.57 µM (micromolar) for lupeol and −7.51 kcal/mol and 3.15 µM (micromolar) for ψ-taraxasterol, respectively. The analysis identified four alkyl bonds, two pi–alkyl bonds, and eight van der Waals interactions between SMAD5 and lupeol. Conversely, the SMAD5 and ψ-taraxasterol interactions comprised five alkyl bonds, one pi–alkyl bond, three van der Waals interactions, and one conventional hydrogen bond. Importantly, the most prominent interaction among these bonds was observed at the Pro96 residue, characterized by a conventional hydrogen bond length of 2.23 Å (Figure S7).

Finally, the interactions between the TGF-β1 ligand and the compounds lupeol and ψ-taraxasterol were investigated. The lowest binding energy and estimated inhibition constant values were determined to be −8.33 kcal/mol and 785.51 nM (nanomolar) for lupeol and −8.85 kcal/mol and 327.35 nM (nanomolar) for ψ-taraxasterol, respectively. The study uncovered two conventional hydrogen bonds, six alkyl bonds, one pi–alkyl bond, and eight van der Waals interactions between TGF-β1 and lupeol. Importantly, significant interactions were observed at the Tyr39 and Met104 residues, with bond lengths of 2.06 Å and 1.86 Å, respectively. Conversely, the interaction between TGF-β1 and ψ-taraxasterol displayed nine alkyl bonds, one pi–alkyl bond, and six van der Waals interactions (Figure S8).

### 3.3. Lupeol and ψ-Taraxasterol Modulate SMAD-Responsive Reporter Activities

**Figure 6:**
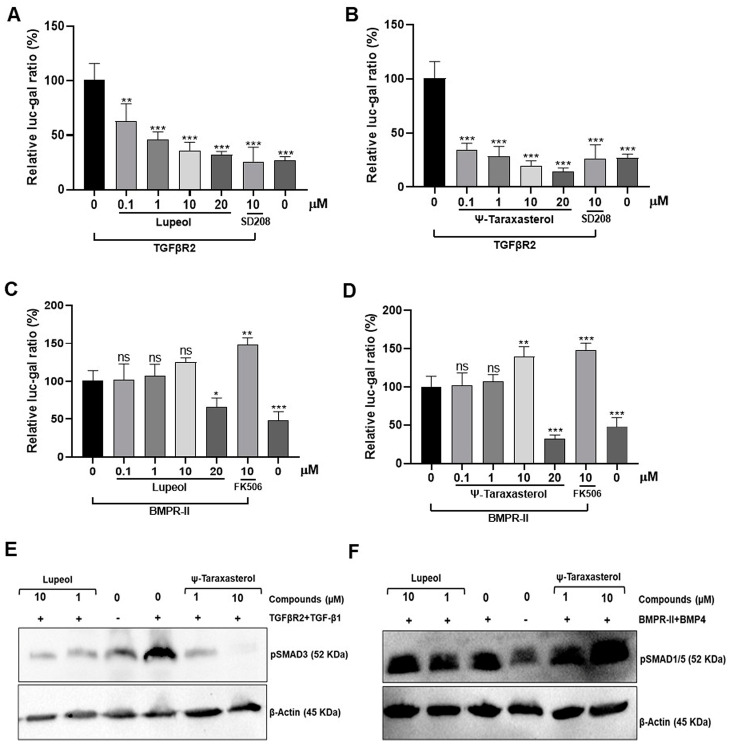
Relative luc-gal rate of lupeol (**A**) and ψ-taraxasterol (**B**) in HEK293T cells transfected with TGFβR2 (100 ng/mL) at concentrations of 0.1 µM, 1 µM, 10 µM, and 20 µM, as well as positive control of SD208 (10 µM), a recognized ALK5 inhibitor. Relative luc-gal rate of lupeol (**C**) and ψ-taraxasterol (**D**) in HEK293T cells transfected with BMPR-II (100 ng/mL) at concentrations of 0.1 µM, 1 µM, 10 µM, and 20 µM, as well as a positive control of FK506 (10 µM), a recognized agent that promotes the BMP pathway. The luc/gal ratio was set as 100 for the TGFBR2- or BMPR-II-overexpressed, untreated group. The figure is representative of SMAD3 phosphorylation in TGFβR2 (100 ng/mL)-overexpressed and TGF-β1 (10 ng/mL)-stimulated HEK 293T cells detected by Western blotting in the presence of lupeol (1 and 10 µM) and ψ-taraxasterol (1 and 10 µM) (**E**) and SMAD1/5 phosphorylation in BMPR-II (100 ng/mL)-overexpressed and BMP4 (10 ng/mL)-stimulated HEK 293T cells detected by Western blotting in the presence of lupeol (1 and 10 µM) and ψ-taraxasterol (1 and 10 µM) (**F**). The means ± standard deviations of three independent experiment sets of data are represented. GraphPad Prism 9.4.0 (453) and APA (ns 0.12, * 0.033, ** 0.002, *** <0.001), compared with the untreated group.

The modulatory effects of lupeol and ψ-taraxasterol on TGF-β, identified through in silico analyses, were verified by employing SMAD-responsive reporter activity in HEK293T cells (database: Cellosaurus; accession number: CVCL_0063). The validity of SMAD3-responsive reporter activity was confirmed by the enhanced reporter activity observed in response to TGFBR2 overexpression (Figure 6A). Then, the modulatory effects of lupeol and ψ-taraxasterol on TGF-β pathways were determined. Lupeol and ψ-taraxasterol caused dose-dependent inhibition of TGF-β-responsive reporter activity, as determined by the luc-gal reporter assay in HEK293T cells overexpressed with the TGFβR2 receptor (Figure 6A,B).

To determine the regulatory effects of lupeol and ψ-taraxasterol on the BMP pathway, SMAD1/5-responsive reporter activity was measured in HEK293T cells. BMPR-II overexpression resulted in SMAD-responsive reporter activity in HEK293T cells, supporting the validity of the method used (Figure 6C,D). Lupeol and ψ-taraxasterol did not exhibit a significant effect for the concentration ranges of 0.01 and 10 µM on the BMP pathway. Furthermore, 0.01–1 µM of ψ-taraxasterol did not promote the BMP-responsive reporter activity. However, 10 µM of ψ-taraxasterol displayed significant promotion for the BMP pathway (Figure 6C,D). Treatment of cells with 10 µM of ψ-taraxasterol increased the reporter activity, an effect likely to be attributed to the activation of SMAD1/5 dependentpathway. Treatment with 20 µM of lupeol and ψ-taraxasterol generated fewer cells compared with the control concentrations, suggesting that this concentration might be toxic to the cells (Figure 6C,D).

### 3.4. Lupeol and ψ-Taraxasterol Reduced the Phosphorylation of SMAD3 and Increased SMAD1/5 Proteins as Determined by Western Blotting

In line with the existing data, our study showed that overexpression of TGFBR2 and stimulation of TGF-β1 resulted in an increase level of phosphorylation of SMAD3 protein, whereas overexpression of BMPR-II and stimulation of BMP4 induced the phosphorylation of SMAD1/5 levels (Figure 6E,F). Data derived from this study suggest that lupeol and ψ-taraxasterol reduce the level of phosphorylation of SMAD3 protein in cells overexpressed with the TGFBR2 receptor and stimulated with the TGF-β1 ligand (Figure 6E and Figure S8). Furthermore, cells treating with 10 µM of ψ-taraxasterol were able to increase the phosphorylation of SMAD 1/5 protein following overexpression of BMPR-II receptor and BMP4 ligand stimulation (Figure 6F and Figure S7).

### 3.5. Lupeol and ψ-Taraxasterol Modulate the Expression of Target Genes Determined by RT-PCR and Q-PCR Analyses

**Figure 7:**
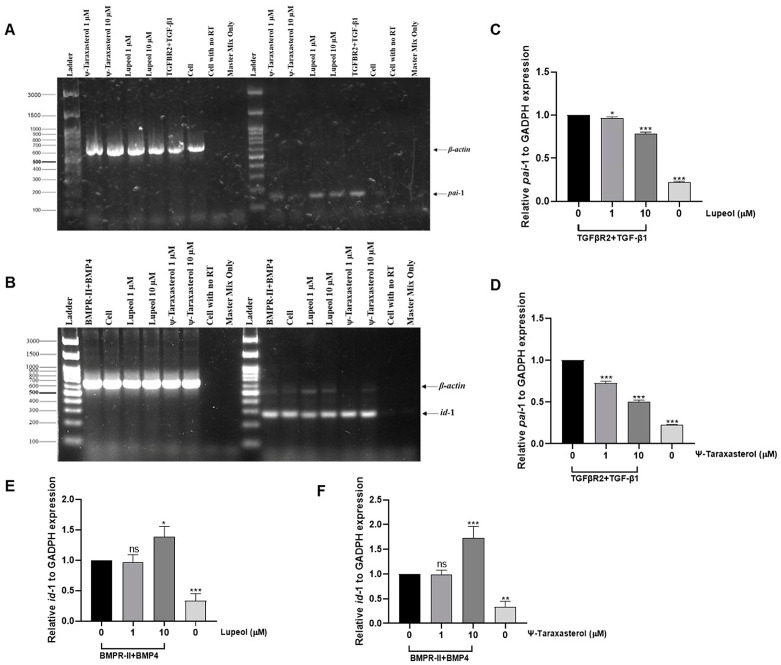
Representative of *β-actin* and *pai*-1 gene expression for lupeol (1 and 10 µM) and ψ-taraxasterol (1 and 10 µM) in TGFβR2 (100 ng/mL)-overexpressed and TGF-β1 (10 ng/mL)-stimulated HEK 293T cells investigated by RT-PCR analysis (**A**) and *β-actin* and *id*-1 gene expression for lupeol (1 and 10 µM) and ψ-taraxasterol (1 and 10 µM) in BMPR-II (100 ng/mL)-overexpressed and BMP4 (10 ng/mL)-stimulated HEK 293T cells detected by RT-PCR analysis (**B**). Quantitative gene expression levels of *pai*-1 for lupeol (1 and 10 µM) (**C**) and ψ-taraxasterol (1 and 10 µM) (**D**) in TGFβR2 (100 ng/mL)-overexpressed and TGF-β1 (10 ng/mL)-stimulated HEK 293T cells detected by Q-PCR analysis. Quantitative gene expression levels of *id*-1 for lupeol (1 and 10 µM) (**E**) and ψ-taraxasterol (1 and 10 µM) (**F**) in BMPR-II (100 ng/mL)-overexpressed and BMP4 (10 ng/mL)-stimulated HEK 293T cells detected by Q-PCR analysis. The means ± standard deviations of three independent experiment sets of data are represented. GraphPad Prism 9.4.0 (453) and APA (ns 0.12, * 0.033, ** 0.002, *** < 0.001).

To determine the effects of the compounds on target gene expression, RT-PCR analysis was performed in HEK293T cells overexpressed with the TGFBR2 receptor and stimulated with the TGF-β1 ligand. RT-PCR analysis confirmed the inhibitory effects of lupeol and ψ-taraxasterol for pai-1 gene expression, a recognized target of the TGF-β pathway. Lupeol and ψ-taraxasterol downregulated the expression of pai-1 in TGFβR2 (100 ng/mL)-overexpressed and TGF-β1 (10 ng/mL)-stimulated HEK293T cells at concentrations of 1 µM and 10 µM. The highest inhibitory effect was observed for 10 µM of ψ-taraxasterol (Figure 7A). RT-PCR analysis for the BMP pathway was conducted in BMPR-II-overexpressed and BMP4-stimulated HEK293T cells. Lupeol, at both concentrations tested, did not induce a significant effect for id-1 gene expression, a recognized target of the BMP pathway. However, ψ-taraxasterol showed a significant stimulatory effect for id-1 gene expression, especially at 10 µM concentrations (Figure 7B). Similarly to RT-PCR analysis, Q-PCR results demonstrated that lupeol and ψ-taraxasterol had inhibitory effects on pai-1 expression, particularly at 10 µM in the TGF-β pathway (Figure 7C,D). For the BMP pathway, 1 µM of both compounds did not exhibit a meaningful effect for id-1 expression in *BMPR2* (100 ng/mL)-overexpressed and BMP4 (10 ng/mL)-stimulated HEK 293T cells, as determined by Q-PCR analysis (Figure 7E,F). Then, 1 µM of ψ-taraxasterol induced moderate promotion of id-1 expression; however, at 10 µM, the compound elicited a very strong effect on id-1 expression (Figure 7F).

### 3.6. Lupeol and ψ-Taraxasterol Reduced Excessive Cell Proliferation of PASMCs Harboring a Pathogenic BMPR2 Mutation

**Figure 8:**
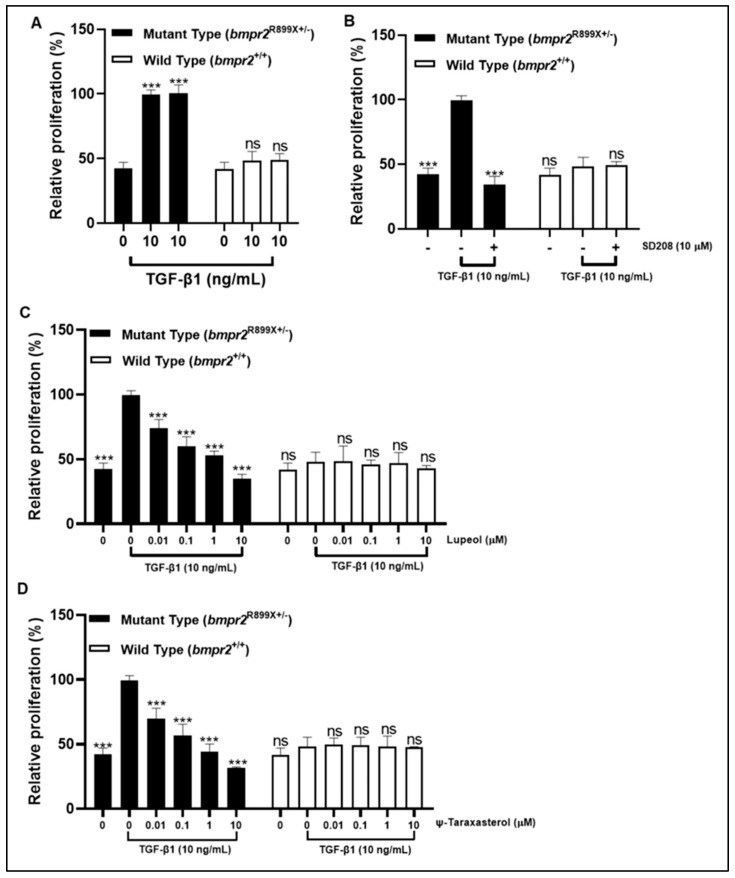
Relative proliferation rate in TGF-β1 (10 ng/mL)-stimulated mutant-type PAMSCs (*bmpr2*^R899X(+/-)^) and wild-type PAMSCs (*bmpr2*^+/+^) in the presence of TGF-β1 (10 ng/mL) (**A**); positive control of SD208 (10 µM) (**B**); lupeol (0.01–10 µM) (**C**); and ψ-taraxasterol (0.01–10 µM) (**D**). GraphPad Prism 9.4.0 (453), APA (ns 0.12 (*** < 0.001)).

TGF-β1 (10 ng/mL) stimulation increased the level of cell proliferation of mutant-type (*bmpr2*^R899X+/-^) PAMSCs, while it did not elicit any significant effect on wild-type (*bmpr2*^+/+^) PAMSCs (Figure 8A). SD208, a recognized inhibitor of ALK5 receptor, had an inhibitory effect on mutant-type PAMSCs (*bmpr2*^R899X+/-^) (Figure 8B). Lupeol and ψ-taraxasterol displayed dose-dependent antiproliferative effects in TGF-β1-stimulated mutant-type PAMSCs (*bmpr2*^R899X+/-^) at concentrations of 0.1–10 µM (Figure 8C,D). However, both compounds induced no significant effect on proliferation in wild-type (*bmpr2*^+/+^) PAMSCs at all concentrations (Figure 8C,D).

## 4. Discussion

**Figure 9:**
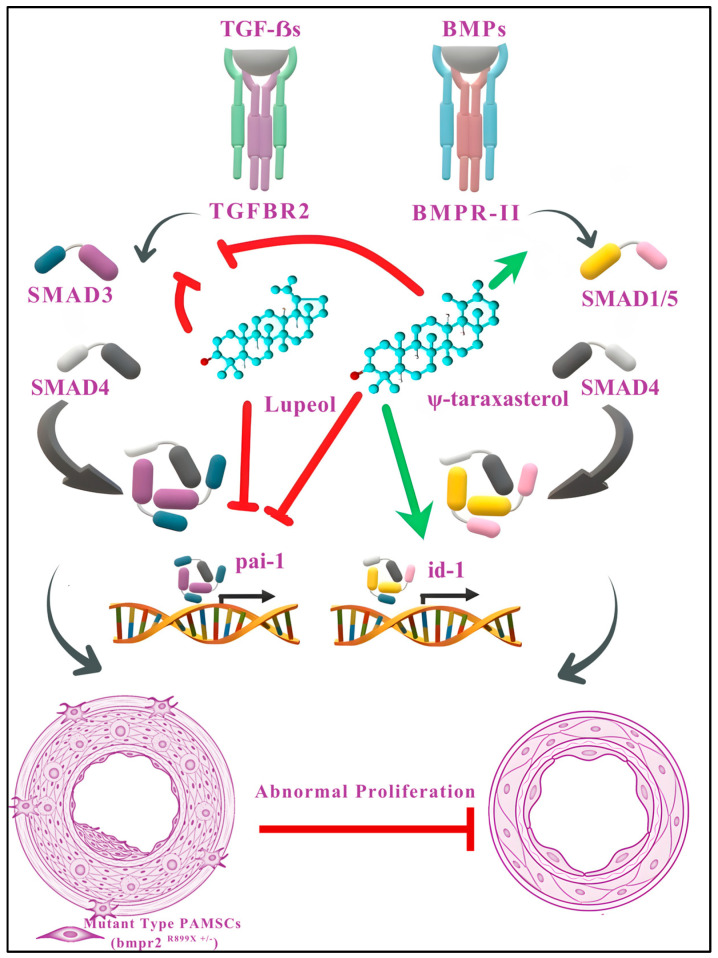
Proposed mechanistic model illustrating the modulatory effects of lupeol and ψ-taraxasterol on TGF-β and BMP signaling pathways. Lupeol and ψ-taraxasterol regulate the TGF-β and/or BMP signaling cascades to counteract abnormal proliferation in pulmonary artery smooth muscle cells (PASMCs). Both compounds suppress TGF-β-mediated signaling by inhibiting the phosphorylation of SMAD3 and reducing the expression of *pai*-1 transcripts, thereby attenuating profibrotic and proliferative responses. Furthermore, ψ-taraxasterol enhances BMP signaling by promoting the phosphorylation of SMAD1/5 and upregulating *id*-1 expression, which supports antiproliferative and homeostatic functions. Collectively, these actions mitigate the aberrant proliferation observed in mutant-type (*bmpr2*^R899X+/-^) PASMCs without exerting cytotoxic effects on wild-type cells. Green arrows indicate stimulation or upregulation; red arrows denote inhibition or downregulation. The images of blood vessels have been adapted from Gayatri and Rajagopal, 2023 [44].

In this study, four major phytoconstituents were isolated, including two flavonoids—luteolin-7-O-β-D-glucopyranoside (CS-A1) and apigenin (CS-E1)—and two triterpenoids—ψ-taraxasterol (CS-C1) and lupeol (CS-C2). Although all four compounds were successfully characterized, subsequent mechanistic and functional assays primarily focused on the triterpenoids ψ-taraxasterol and lupeol, which were evaluated to determine their potential modulatory effects on BMP and TGF-β signaling pathways and their ability to restore cellular defects in PASMCs harboring a pathogenic *BMPR2* mutation. We demonstrated a number of ways that lupeol and ψ-taraxasterol inhibited the TGF-β signaling pathway by reducing the phosphorylation of SMAD3 protein and the expression of pai-1 transcripts (Figure 9). Additionally, ψ-taraxasterol enhanced BMP signaling via regulating the phosphorylation of SMAD1/5 proteins and increased the expression of id-1 transcripts. Finally, both lupeol and ψ-taraxasterol inhibited abnormal proliferation of mutant-type (*bmpr2*^R899X+/-^) PAMSCs with no discernible cytotoxic effects on wild-type cells. This is the first comprehensive report outlining the anti-TGF-β and pro-BMP effects of lupeol and ψ-taraxasterol, which upon further validation may have preclinical and clinical applications.

These flavonoid and triterpenoid compounds are particularly noteworthy in the context of pulmonary arterial hypertension (PAH), as they target critical mechanisms underlying disease progression. Flavonoids such as luteolin derivatives and apigenin have been shown to attenuate oxidative stress and suppress pro-inflammatory signaling pathways, thereby ameliorating endothelial dysfunction and vascular remodeling, which are considered central features of PAH pathophysiology [45,46,47,48]. In parallel, triterpenoids such as lupeol have been extensively reported to exert vasoprotective and antiproliferative effects. Although taraxasterol has been reported to exert beneficial effects, such as anti-inflammatory and antioxidant activities that may be associated with the treatment of cardiovascular disorders, studies specifically addressing the pharmacological potential of ψ-taraxasterol remain rather limited [49,50,51]. These findings highlight flavonoid and triterpenoid compounds as promising candidates for developing novel therapeutic strategies against PAH. Since the pharmacological effects of lupeol and apigenin in PAH have been extensively investigated in previous studies, the present work was therefore confined to two triterpenoid compounds.

To elucidate the molecular mechanism underlying these effects, the present study investigated whether these triterpenes could modulate key signaling targets involved in PAH. We and others previously demonstrated that dysregulation of TGF-β and BMP signaling pathways contributes significantly to the pathogenesis of PAH [52]. Accordingly, in silico analyses were performed to determine whether lupeol and ψ-taraxasterol interact with key components of these signaling cascades. Computational modeling, as an integral part of modern drug discovery, provides a rapid and cost-effective approach to predict ligand–target interactions and identify promising candidates prior to biological validation [53,54,55]. Consistent with this rationale, our simulations revealed that both triterpenes were capable of engaging multiple nodes within the TGF-β/BMP axis. Notably, molecular docking analyses revealed that both lupeol and ψ-taraxasterol exhibited dual receptor-binding potential, showing strong affinities toward the BMPR-II and TGFBR2 receptors. In addition to receptor-level interactions, these triterpenoids also engaged with several downstream signaling mediators, including SMAD1, SMAD3, SMAD4, SMAD5, and the TGF-β1 ligand, suggesting that their modulatory effects may arise from multi-target interactions across both the TGF-β and BMP signaling pathways. These computational results support the hypothesis that both triterpenes may act as modulators of the TGF-β and BMP signaling pathways, which are critically involved in vascular remodeling processes underlying PAH.

To further validate these computational predictions and substantiate their biological relevance, cell-based reporter assays were conducted to assess the impact of these compounds on TGF-β and BMP signaling activities. Our findings suggest that both lupeol and ψ-taraxasterol may restrict TGF-β activity through SMAD3-dependent pathways (Figure 9). This was supported by cell-based TGF-β-responsive reporter assay data, which demonstrated a dose-dependent inhibition of TGF-β signaling by both compounds. Given the detrimental role of aberrant TGF-β activation in PAH, often linked to irregular TGF-β expression [56], the ability of these triterpenes to modulate this pathway highlights their potential as therapeutic candidates. Moreover, patients with hereditary PAH typically exhibit reduced BMPR-II expression in lung tissue, decreased plasma BMP activity, and pathological hemodynamic parameters such as elevated pulmonary vascular resistance and right ventricular hypertrophy [57]. Importantly, our BMP-responsive reporter assay revealed that ψ-taraxasterol enhanced BMP signaling, a mechanism that is considered a promising therapeutic target in PAH [56]. Thus, ψ-taraxasterol, by restoring impaired BMP signaling, may represent a valuable phytochemical source for the development of novel treatment strategies against PAH.

To validate the findings derived from *in silico* modeling in a reliable, controllable, and cell-based model, BMP- and TGF-β-responsive reporter activities were detected in HEK293T cells, which are widely employed for mechanistic studies of TGF-β and BMP signaling in PAH due to their well-defined genotype and high transfection efficiency [6,10,58,59,60,61,62]. This system offers a reproducible and accepted platform for preliminary screening and receptor-level target validation related to BMPR2-mediated signaling [6,10,63,64].

The TGFBR2 and BMPR-II receptors act as central regulators of downstream SMAD signaling, whose dysregulation is closely linked to PAH pathogenesis, particularly through impaired BMP-SMAD1/5/8 activity and excessive activin–SMAD2/3 responses leading to vascular remodeling [65,66,67]. Our results showed that both lupeol and ψ-taraxasterol modulate SMAD-dependent TGF-β signaling, with ψ-taraxasterol uniquely enhancing BMP-SMAD1/5 signal transduction. Phosphorylation of SMAD2/3 is known to drive PAI-1 upregulation, a characteristic feature of TGF-β–SMAD2/3 activation in PAH [68]. However, our experiments revealed that both compounds suppressed PAI-1 expression in TGFBR2-overexpressing or TGF-β1-stimulated cells, suggesting the anti-TGF β properties of these compounds. In contrast, BMP signaling exerts protective effects through the induction of Id proteins, particularly Id-1, whose expression in PASMCs is mediated via BMP-SMAD1/5 signaling and is diminished by BMPR-II mutations [19]. Cell-based studies indicated that ψ-taraxasterol upregulated Id-1 expression, suggesting its pro-BMP-SMAD1/5 activity.

To determine whether these anti-TGF β and pro-BMP effects rescued cellular defects, we further evaluated the impact of lupeol and ψ-taraxasterol on the rate of proliferation of PASMCs harboring a pathogenic *bmpr2* mutation (Figure 9). Using both mutant (*bmpr2*^R899X+/-^) and wild-type (*bmpr2*^+/+^) mouse PASMCs in an MTT assay, we observed that the *bmpr2*^R899X^ mutation—previously linked to the hyperproliferative phenotype of PAH—exhibited enhanced proliferation upon TGF-β stimulation, consistent with earlier reports [69,70,71]. Notably, both compounds produced dose-dependent antiproliferative effects in TGF-β1-stimulated mutant PASMCs, while no cytotoxicity was detected in wild-type cells. The lack of growth suppression effects in healthy PASMCs suggests that compounds selectively target the excessive rate of proliferation of mutant cells, highlighting lupeol and ψ-taraxasterol as promising disease-modifying agents that address the underlying cause of vascular pathology of PAH.

Given these antiproliferative properties, the safety profiles of lupeol and ψ-taraxasterol were also considered to define their potential therapeutic window. Current evidence provides key preliminary insights: lupeol exhibits dose-dependent antiproliferative effects in cancer cells (IC_50_ = 20–80 µM), while normal fibroblasts and epithelial cells show minimal cytotoxicity at ≤10 µM and only moderate effects at higher doses (>50–100 µM) [72]. Comparable data for ψ-taraxasterol are limited; studies on its structural isomer taraxasterol indicate similar tumor cell cytotoxicity, with low toxicity in non-transformed cells [73]. Literature reports describe lupeol as a nontoxic compound in animals at 30–2000 mg/kg [27], whereas no systematic in vivo toxicological evaluation exists for ψ-taraxasterol. In our experiments, neither compound showed cytotoxicity in wild-type PASMCs at 1–10 µM, and in mutant PASMCs, antiproliferative activity appeared at 10 µM without toxicity at 1 µM. These findings suggest a preliminary therapeutic window within the lower micromolar range, although in vivo validation will be required to confirm their clinical relevance.

Whilst the experiments conducted in HEK293T cells provided valuable mechanistic insight into TGF-β and BMP signaling, this cell-based model does not fully reflect the disease-specific characteristics of the vascular remodeling processes observed in vivo. To address this limitation, subsequent experiments were performed in more disease-relevant cells, including PASMCs, confirming the modulatory effects of ψ-taraxasterol and lupeol on BMP–SMAD1/5 and TGF-β–SMAD2/3 signaling. While both compounds showed promising effects in both HEK-293T- and PASMC-based models, further validation in in vivo PAH models is required to fully establish their therapeutic potential. In the United Kingdom, in vivo experimentation is subject to strict ethical regulations and is permitted only after sufficient preliminary preclinical evidence has been established [55]. The findings derived from this study thus contribute to fulfilling this prerequisite by providing a strong rationale to support animal investigations in the future.

## 5. Conclusions and Future Perspectives

This study provides preliminary in vitro evidence that ψ-taraxasterol and lupeol exert modulatory effects on signaling pathways central to PAH pathogenesis. In silico analyses suggested that both compounds interact with multiple elements of the TGF-β pathway, while ψ-taraxasterol exhibited stronger associations with BMP-related components. These predictions were supported by functional assays, where both compounds attenuated TGF-β–SMAD2/3-dependent signaling at concentrations ranging from 0.1 to 10 µM. For the BMP pathway, ψ-taraxasterol enhanced SMAD1/5 phosphorylation, particularly at 10 µM, whereas lupeol showed no clear stimulatory effect. In cell proliferation assays, both compounds produced dose-dependent antiproliferative effects in mutant PASMCs (*bmpr2*^R899X+/-^), but not in wild-type PASMCs (*bmpr2*^+/+^), suggesting a degree of selectivity for disease-relevant cells. The absence of observable effects in wild-type cells may also indicate a favorable preliminary safety profile. Despite these promising in vitro findings, the absence of in vivo validation represents a major limitation. Without animal model studies, it remains uncertain whether these compounds achieve sufficient bioavailability, maintain activity in the complex physiological context, or exhibit an acceptable therapeutic window. Therefore, while ψ-taraxasterol and lupeol appear to be potential candidates for modulating pathogenic PASMC signaling, these observations should be considered exploratory.

Future work should build upon these findings by elucidating the structure–activity relationship (SAR) and assessing the efficacy, selectivity, and potency of the compounds. In addition, molecular dynamics simulations could provide deeper insight into binding stability and conformational flexibility. Complementary biophysical assays using purified targets would help determine binding affinity. Once adequate evidence is obtained, subsequent investigations using experimental PAH models could clarify whether ψ-taraxasterol and lupeol offer therapeutic intervention prior to or following the onset of PAH.

## Data Availability

The original contributions presented in this study are included in the article/Supplementary Material. Further inquiries can be directed to the corresponding author.

## Supplementary Materials

The following supporting information can be downloaded at https://www.mdpi.com/article/10.3390/cimb47110939/s1. Figure S1. Schematic diagram of isolation procedure. Figure S2. ^1^H-NMR spectrum (CD3OD, 400 MHz) of Luteolin-7-O-*β*-D-glucopyranoside (CS-A1). Figure S3. (A) ^1^H-NMR spectrum (CD_3_OD, 400 MHz) of Apigenin (CS-E1), (B) ^13^C-NMR spectrum (CD_3_OD, 100 MHz) of Apigenin (CS-E1). Figure S4. (A) ^1^H-NMR spectrum (CDCl_3_, 400 MHz) of *ψ*-taraxasterol (CS-C1), (B) ^13^C-NMR spectrum (CDCl_3_, 100 MHz) of *ψ*-taraxasterol (CS-C1). Figure S5. (A) ^1^H-NMR spectrum (CDCl_3_, 400 MHz) of Lupeol (CS-C2), (B) ^13^C-NMR spectrum (CDCl_3_, 100 MHz) of Lupeol (CS-C2). Figure S6. Binding poses predicted by the BIOVIA Discovery Studio Visualizer with the target SMAD4 extracellular domain receptor. (a) 3D view and (b) 2D ligand interactions of Lupeol; (c) 3D view and (d) 2D ligand interactions of ψ-taraxasterol. Figure S7. Binding poses predicted by the BIOVIA Discovery Studio Visualizer with the target SMAD5 extracellular domain receptor. (a) 3D view and (b) 2D ligand interactions of Lupeol; (c) 3D view and (d) 2D ligand interactions of ψ-taraxasterol. Figure S8. Binding poses predicted by the BIOVIA Discovery Studio Visualizer with the target TGF-β1 extracellular domain receptor. (a) 3D view and (b) 2D ligand interactions of Lupeol; (c) 3D view and (d) 2D ligand interactions of ψ-taraxasterol.

## Abbreviations

βGAL	Beta-galactosidase (reporter gene)
BMPR-II	Bone Morphogenetic Protein Receptor Type II
BMP	Bone Morphogenetic Protein
CD_3_OD	Deuterated Methanol (solvent for NMR spectroscopy)
CDCl_3_	Deuterated Chloroform (solvent for NMR spectroscopy)
CS-A1	Luteolin-7-O-β-D-glucopyranoside
CS-C1	ψ-Taraxasterol
CS-C2	Lupeol
CS-E1	Apigenin
id-1	Inhibitor of DNA Binding 1 (BMP target gene)
MS	Mass Spectrometry
NMR	Nuclear Magnetic Resonance Spectroscopy
NO	Nitric Oxide
PAH	Pulmonary Arterial Hypertension
pai-1	Plasminogen Activator Inhibitor-1 (TGF-β target gene)
PAMSCs	Pulmonary Artery Smooth Muscle Cells
PH	Pulmonary Hypertension
qPCR	Quantitative Polymerase Chain Reaction
R-SMADs	Receptor-regulated SMADs (SMAD1/2/3/5 proteins)
RT-PCR	Reverse Transcription Polymerase Chain Reaction
SBE-LUC	SMAD Binding Element–Luciferase Reporter Assay
TGF-β	Transforming Growth Factor Beta
TGFBR2	Transforming Growth Factor Beta Receptor 2
