# Supplementary material for: Regulation of TGF-β and BMP Signaling by Natural Triterpene Compounds in Pulmonary Arterial Hypertension (PAH)
Source: Curr Issues Mol Biol. 2025 Nov 12;47(11):939. doi: 10.3390/cimb47110939 (PMC12650971; doi:10.3390/cimb47110939)
Supplement: Supplementary file 1 [file cimb-47-00939-s001.zip › cimb-3869416-supplementary.pdf]

current issues in molecular biology

Article

Regulation of TGF-β and BMP Signaling by Natural Triterpene Compounds in Pulmonary Arterial Hypertension (PAH)

Legends of Supplementary Materials

Figure S1. Schematic diagram of isolation procedure

Figure S2. ^{1}H-NMR spectrum (CD3OD, 400 MHz) of Luteolin-7-O-*β*-D-glucopyranoside (CS-A1)

Figure S3. (A) ^{1}H-NMR spectrum (CD_3OD, 400 MHz) of Apigenin (CS-E1), **(B)** ^{13}C-NMR spectrum (CD_3OD, 100 MHz) of Apigenin (CS-E1)

Figure S4. (A) ^{1}H-NMR spectrum ($CDCl_3$, 400 MHz) of *ψ*-taraxasterol (CS-C1), **(B)** ^{13}C-NMR spectrum ($CDCl_3$, 100 MHz) of *ψ*-taraxasterol (CS-C1)

Figure S5. (A) ^{1}H-NMR spectrum ($CDCl_3$, 400 MHz) of Lupeol (CS-C2), **(B)** ^{13}C-NMR spectrum ($CDCl_3$, 100 MHz) of Lupeol (CS-C2)

Figure S6. Binding poses predicted by the BIOVIA Discovery Studio Visualizer with the target SMAD4 extracellular domain receptor. a) 3D view and (b) 2D ligand interactions of Lupeol; (c) 3D view and (d) 2D ligand interactions of ψ-taraxasterol

Figure S7. Binding poses predicted by the BIOVIA Discovery Studio Visualizer with the target SMAD5 extracellular domain receptor. a) 3D view and (b) 2D ligand interactions of Lupeol; (c) 3D view and (d) 2D ligand interactions of ψ-taraxasterol

Figure S8. Binding poses predicted by the BIOVIA Discovery Studio Visualizer with the target TGF-β1 extracellular domain receptor. a) 3D view and (b) 2D ligand interactions of Lupeol; (c) 3D view and (d) 2D ligand interactions of ψ-taraxasterol

 https://doi.org/10.3390/cimb47110939

Supplementary Materials

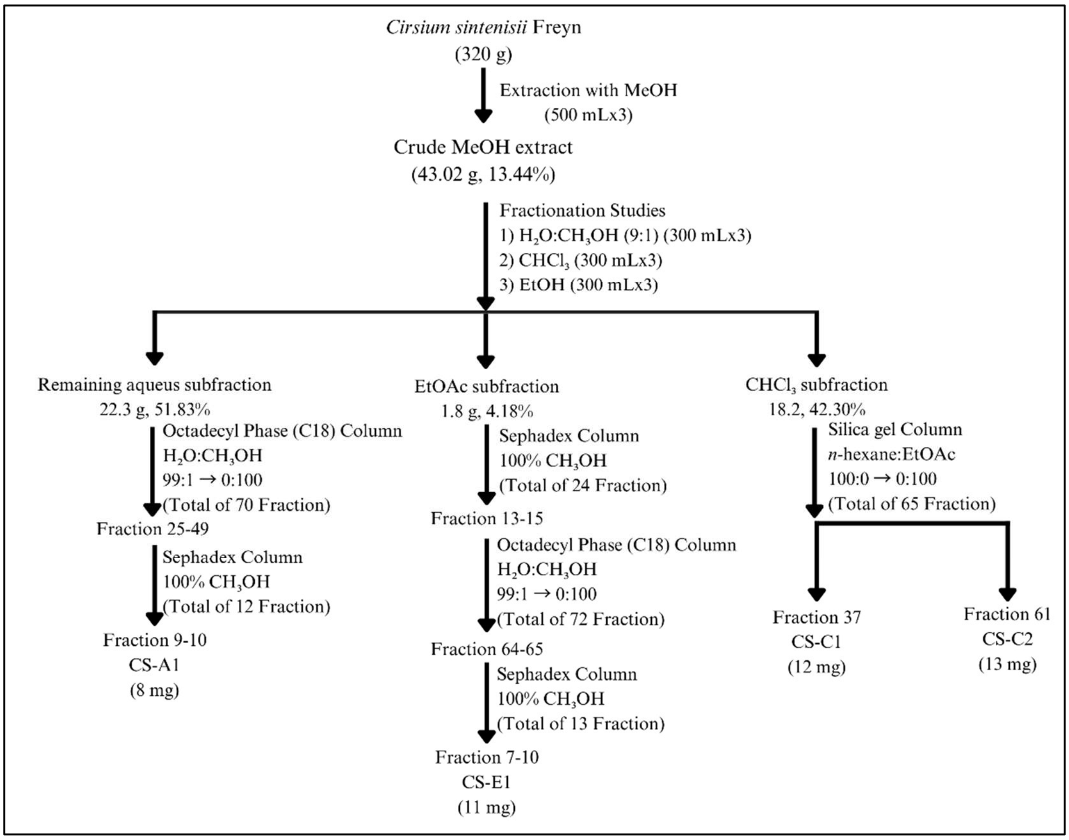

Figure S1. Schematic diagram of isolation procedure

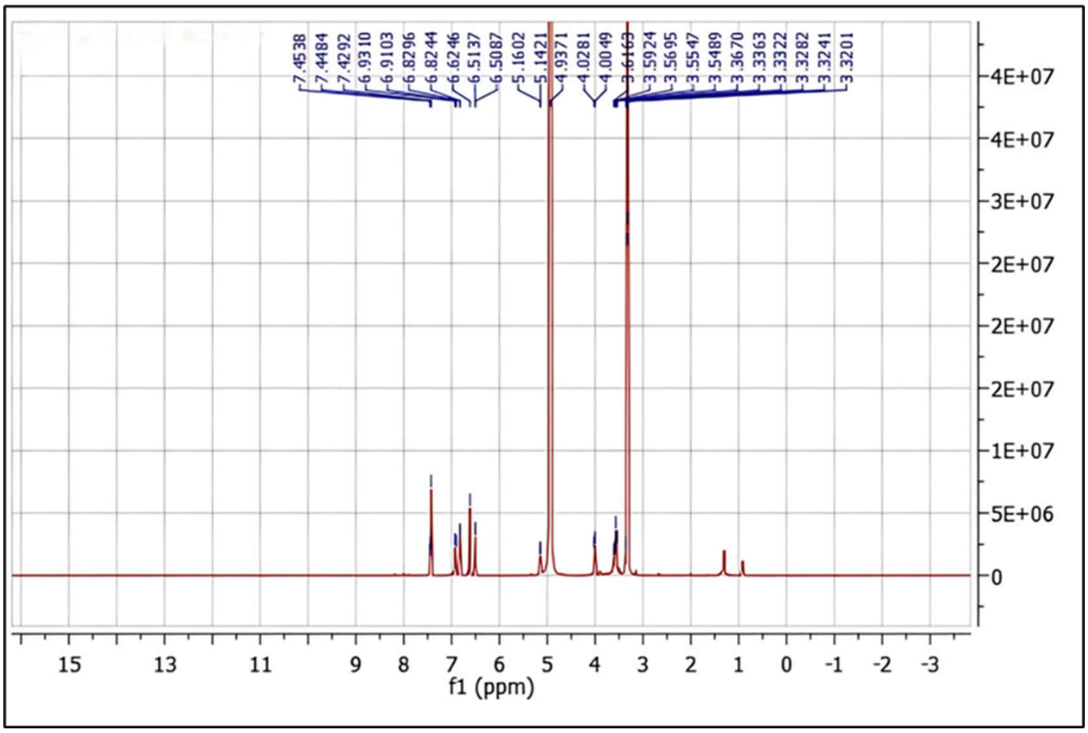

Figure S2. ^{1}H-NMR spectrum (CD_3OD, 400 MHz) of Luteolin-7-O-*β*-D-glucopyranoside (CS-A1)

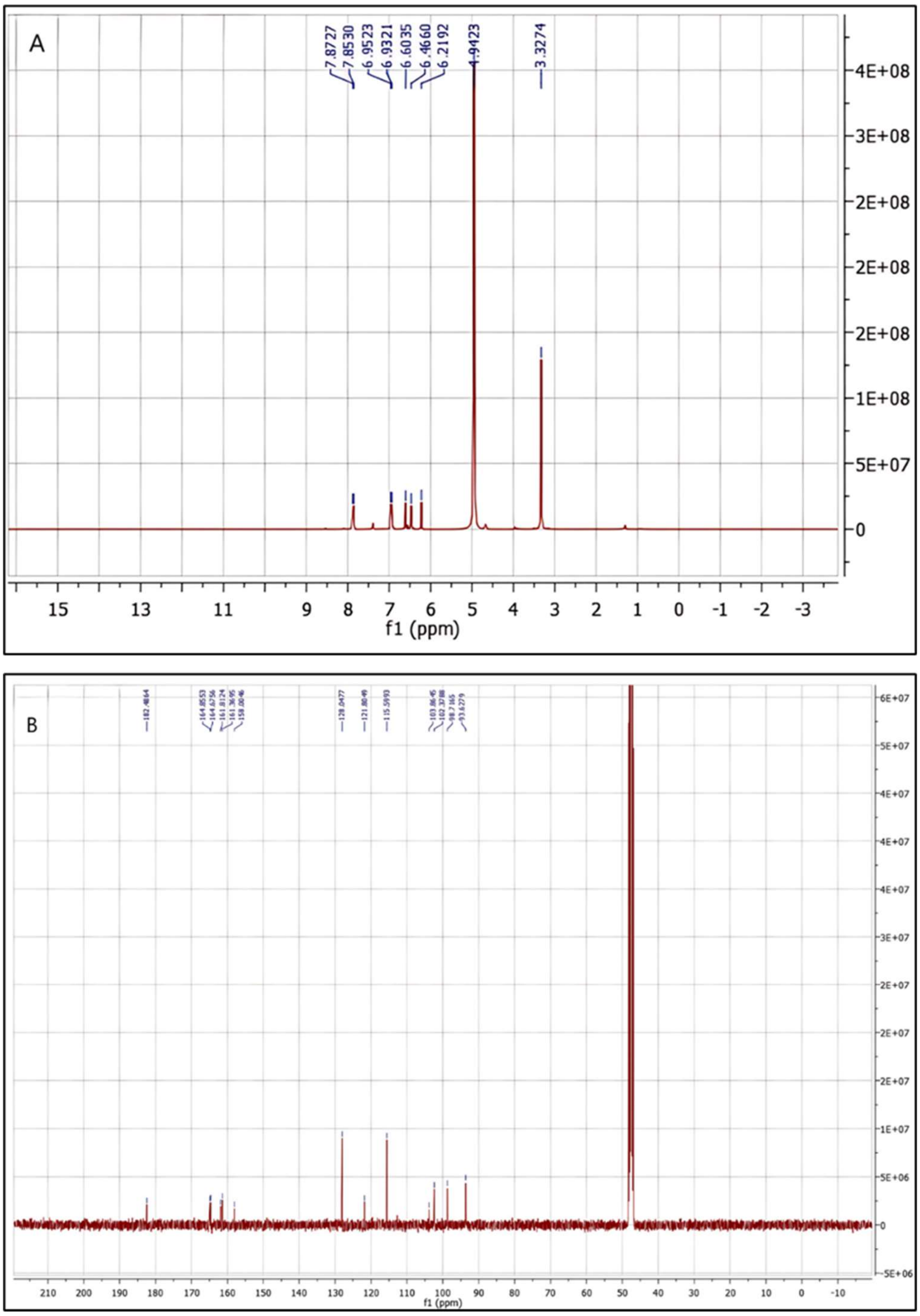

Figure S3. (A) ^{1}H-NMR spectrum (CD_3OD, 400 MHz) of Apigenin (CS-E1), **(B)** ^{13}C-NMR spectrum (CD_3OD, 100 MHz) of Apigenin (CS-E1)

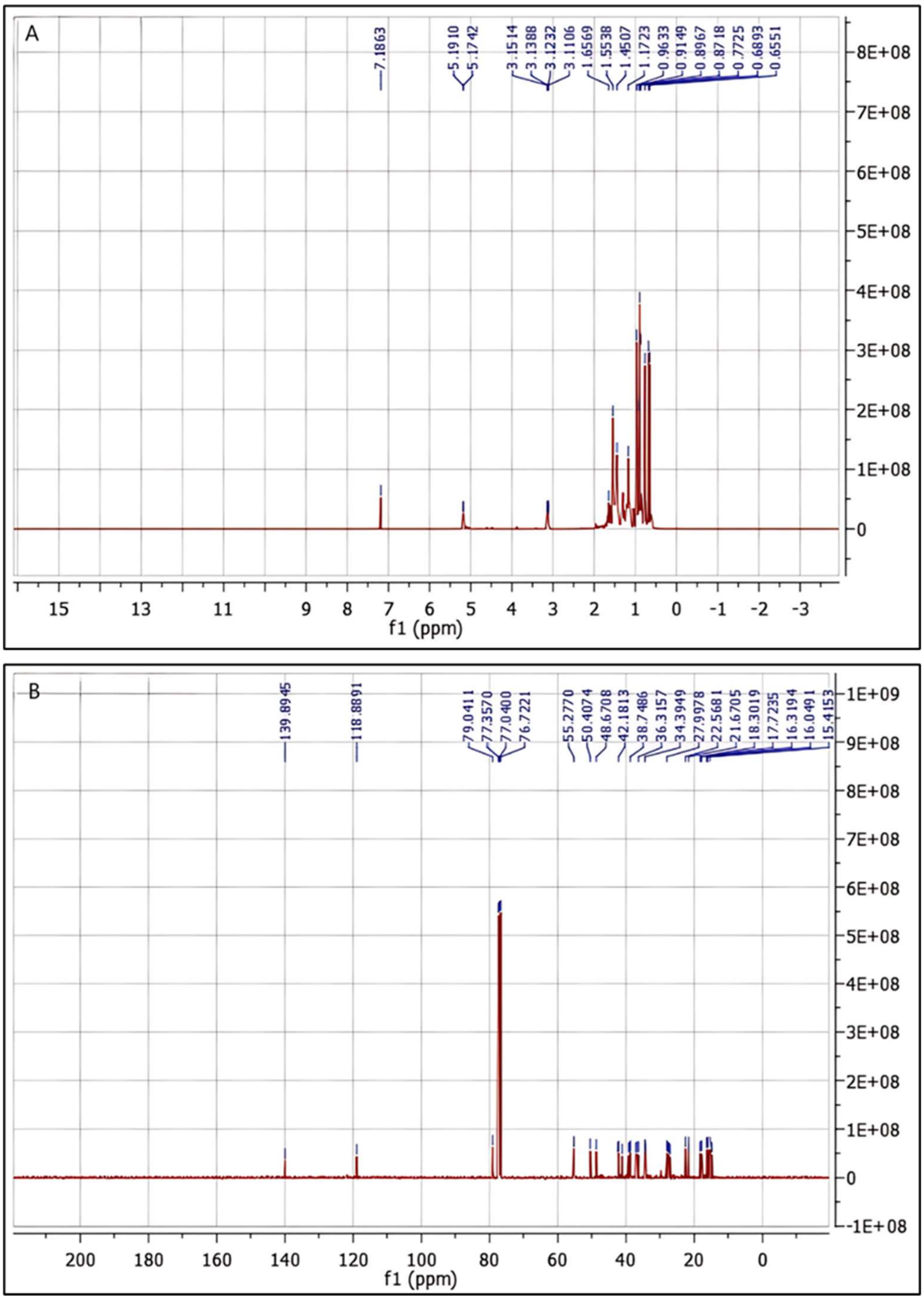

Figure S4. (A) ^{1}H-NMR spectrum ($CDCl_3$, 400 MHz) of *ψ*-taraxasterol (CS-C1), **(B)** ^{13}C-NMR spectrum ($CDCl_3$, 100 MHz) of *ψ*-taraxasterol (CS-C1)

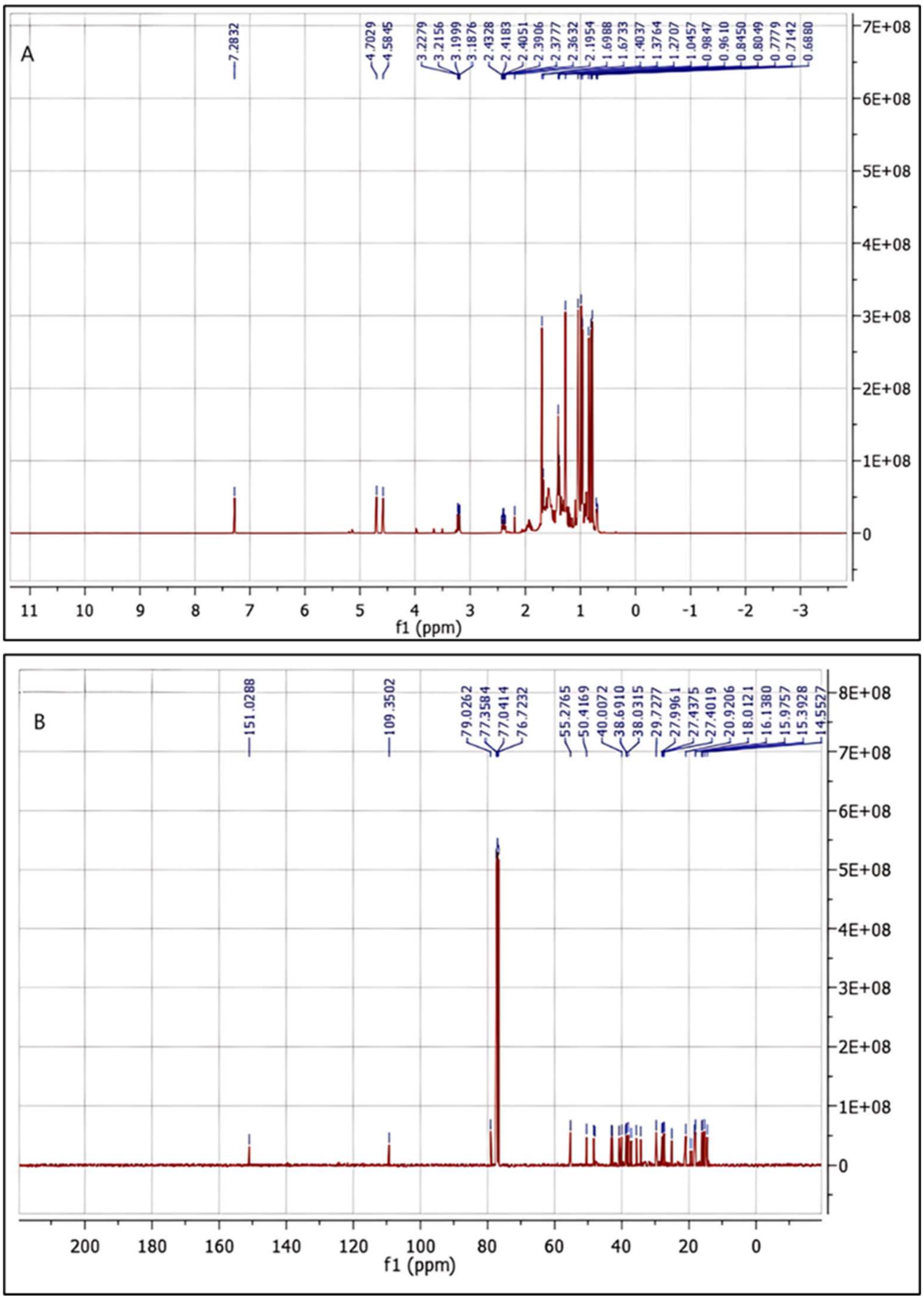

Figure S5. (A) ^{1}H-NMR spectrum ($CDCl_3$, 400 MHz) of Lupeol (CS-C2), **(B)** ^{13}C-NMR spectrum ($CDCl_3$, 100 MHz) of Lupeol (CS-C2)

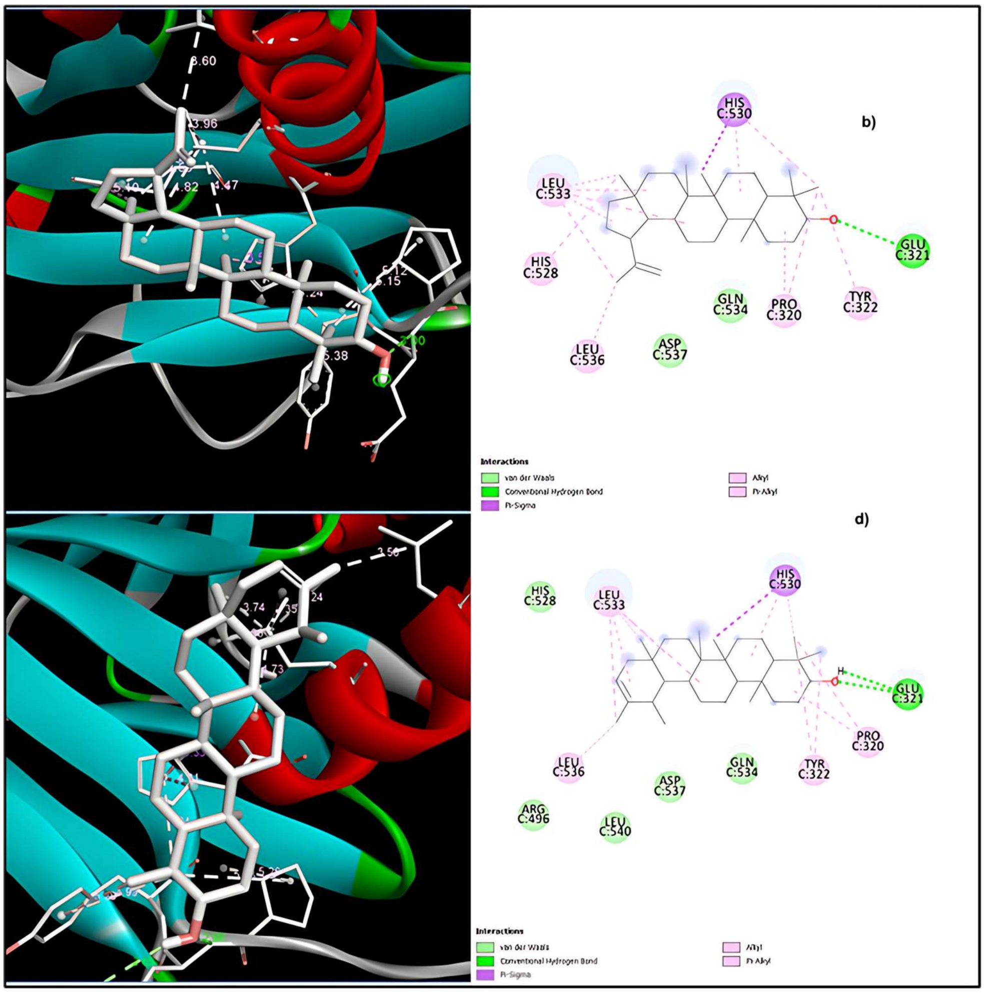

Figure S6. Binding poses predicted by the BIOVIA Discovery Studio Visualizer with the target SMAD4 extracellular domain receptor. (a) 3D view and (b) 2D ligand interactions of Lupeol; (c) 3D view and (d) 2D ligand interactions of ψ-taraxasterol

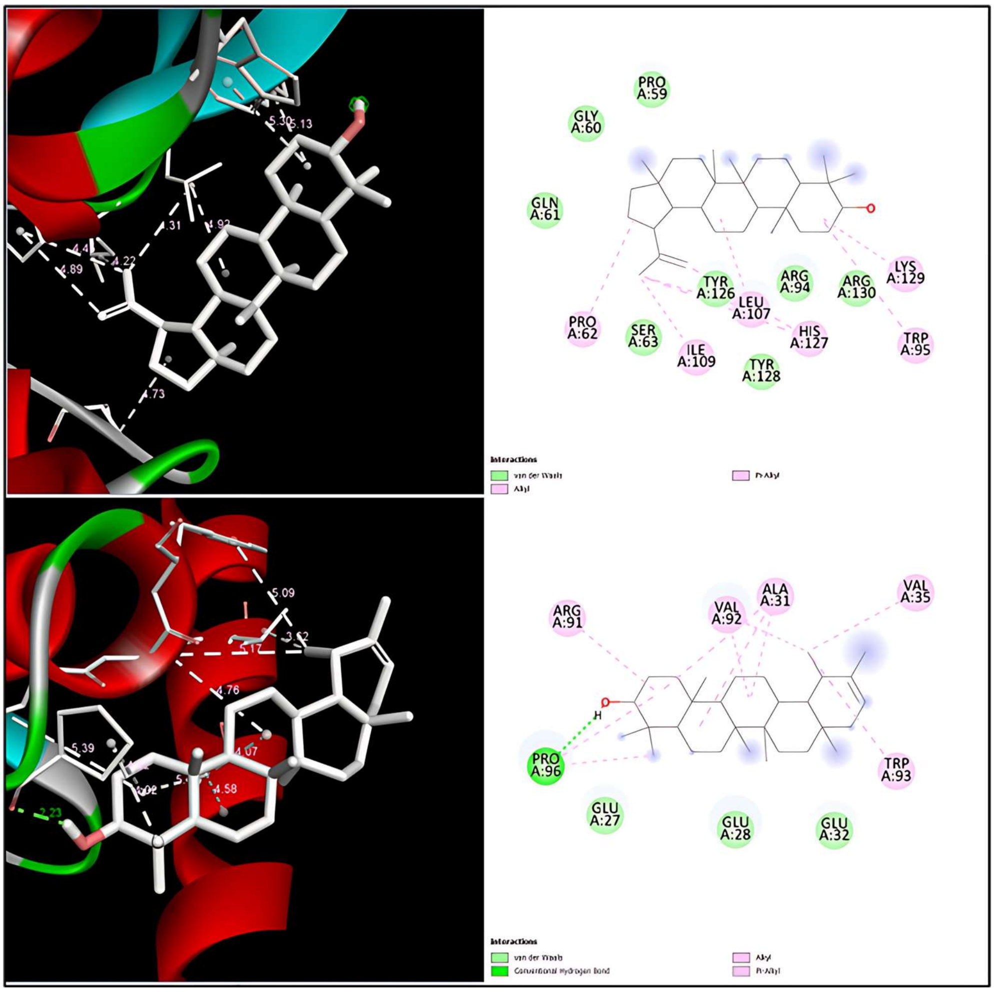

Figure S7. Binding poses predicted by the BIOVIA Discovery Studio Visualizer with the target SMAD5 extracellular domain receptor. (a) 3D view and (b) 2D ligand interactions of Lupeol; (c) 3D view and (d) 2D ligand interactions of ψ-taraxasterol

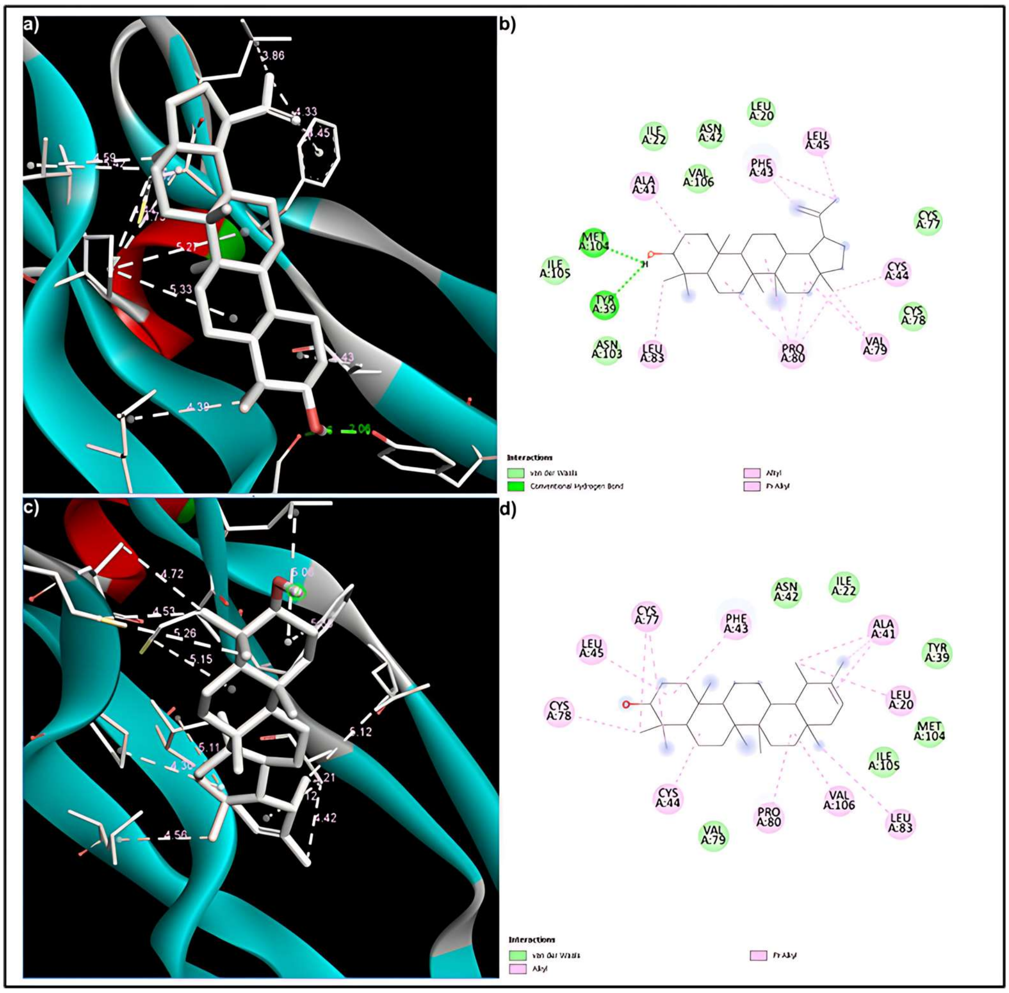

Figure S8. Binding poses predicted by the BIOVIA Discovery Studio Visualizer with the target TGF-β1 extracellular domain receptor. (a) 3D view and (b) 2D ligand interactions of Lupeol; (c) 3D view and (d) 2D ligand interactions of ψ-taraxasterol

Disclaimer/Publisher's Note: The statements, opinions and data contained in all publications are solely those of the individual author(s) and contributor(s) and not of MDPI and/or the editor(s). MDPI and/or the editor(s) disclaim responsibility for any injury to people or property resulting from any ideas, methods, instructions or products referred to in the content.
